# Into the large ears: otitis externa associated with nematodes, mites, and bacteria in Asian elephants (*Elephas maximus*)

**DOI:** 10.1186/s13071-023-05708-1

**Published:** 2023-03-06

**Authors:** Marcos Antonio Bezerra-Santos, Jairo Alfonso Mendoza-Roldan, Peter M. DiGeronimo, Erica Ward, Bruce Noden, Francesca De Luca, Elena Fanelli, Domenico Valenzano, Riccardo Paolo Lia, Domenico Otranto

**Affiliations:** 1 Department of Veterinary Medicine, University of Bari, Valenzano, Bari, Italy; 2 Philadelphia Zoo, Philadelphia, PA USA; 3 Wildlife and Animal Welfare Institute, Wrentham, MA USA; 4 Department of Entomology and Plant Pathology, Oklahoma State University, Oklahoma, USA; 5 Institute for the Sustainable Protection of Plants, National Research Council, Bari, Italy; 6 Department of Soil, Plant and Food Sciences, University of Bari, Bari, Italy; 7 Department of Pathobiology, Faculty of Veterinary Science, Bu-Ali Sina University, Hamedan, Iran

**Keywords:** Elephants, Mites, Nematodes, External otitis, Bacteria, Yeasts, Dust-bathing

## Abstract

**Background:**

The Asian elephant (*Elephas maximus*), which is an endangered species, harbors several parasites. Among the ectoparasites that it harbors, ear mites of the genus *Loxanoetus* have the potential to cause external otitis, an inflammation that may also be associated with the presence of other microorganisms. We assessed the relationships between ear mites, nematodes, yeast, bacterial rods, and cocci sampled from the ears of captive Asian elephants in Thailand. In addition, we discuss the possibility that dust-bathing behavior may be triggered by ear mite infestation, and that this in turn may lead to contamination of the ears with soil microorganisms.

**Methods:**

Legally owned captive Asian elephants (*n* = 64) were sampled. Ear swabs were individually collected from both ears and microscopically examined for the presence of mites, nematodes, yeast, bacterial rods, cocci, and host cells. Mites and nematodes were identified to species level using morphological and molecular methods.

**Results:**

*Loxanoetus lenae* mites were present in 43.8% (*n* = 28/64) of the animals (19 animals with mites in one ear and nine animals with mites in both ears). Nematodes of the genus *Panagrolaimus* were detected in 23.4% (*n* = 15/64) of the animals (10 with nematodes in one ear and five with nematodes in both ears). In adult elephants (Fisher’s exact test, *P* = 0.0278) and female elephants (Fisher’s exact test, *P* = 0.0107), the presence of nematodes in both ears was significantly associated with the presence of mites. In addition, higher categorical burdens of nematodes were also significantly associated with the presence of mites (Fisher’s exact test, *P* = 0.0234) and epithelial cells (Fisher’s exact test, *P* = 0.0108), and marginally significantly associated with bacterial cocci (Fisher’s exact test, *P* = 0.0499).

**Conclusions:**

The presence of *L. lenae* mites in the ear canals of the Asian elephants was significantly associated with the occurrence of other microorganisms, such as soil nematodes, bacteria and yeasts. The presence of mites in their ears may increase the dust-bathing behavior of elephants which, if confirmed, represents a further paradigmatic example of a parasitic infestation affecting animal behavior.

**Graphical Abstract:**

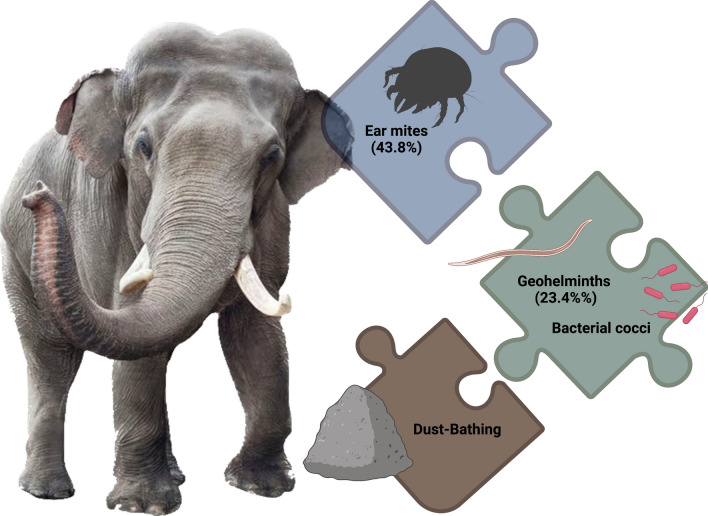

## Background

Asian elephants (*Elephas maximus*) are classified as endangered in the Red List of the International Union for Conservation of Nature [1]. By 2018, the global population of Asian elephants had decreased, and numbered around 48,323–51,680 free-ranging individuals [2] across South and Southeast Asia (i.e., Bangladesh, Bhutan, Cambodia, China, India, Indonesia, Lao, Malaysia, Myanmar, Nepal, Sri Lanka, Thailand, and Vietnam). In addition, around 15,000 individuals were under managed care, mainly in Myanmar, Thailand, Sri Lanka, and India [2].

Elephants harbor endo- (e.g., protozoa, nematodes, trematodes, and cestodes), and ectoparasites (e.g., ticks, mites, lice, fleas, blackflies, mosquitoes, midges, tabanids, and blowflies) that may cause clinical disease depending on the intensity of infection and parasite burden (i.e., degree of parasite load) [3]. As seen in other species, several parasites of elephants that evolved within a host-specific relationship are, in some cases, now extinct due to the decrease in, or extinction of, their specific hosts [4]. For example, the genera *Cobboldia*, *Pharyngobolus*, *Neocuterebra*, and *Ruttenia* of the family Oestridae, as well as several hard tick species, are expected to disappear as the populations of their specific hosts decrease [4, 5]. This may also be the case for the ear mites *Loxanoetus lenae* and *Loxoaneotus bassoni*, which belong to the family Histiotomatidae, and are species-specific parasites of Asian and African elephants, respectively [6, 7]. These mites have the potential to cause external otitis (i.e., inflammation of the ear canal) in their hosts [7], and the affected animals may even present with mucoid discharge from the ear canal requiring specific tests to diagnose the mites’ presence and association with the otitis [7]. In addition, the presence of these parasites in the ear canal may disturb the affected animal, and have an effect on its behavior in the wild by increasing its frequency of dust-bathing; this may lead to the dispersion of soil-borne microorganisms, such as bacteria, and even increase the transmission of ectoparasites, including ear mites, among animals that share the same environment [8].

Ear infestation in captive African elephants (*Loxodonta africana*) by *Loxoaneotus bassoni* mites treated with 5 ml of 1% ivermectin applied to each ear canal every 2 days resulted in the complete elimination of mites 9 days after the first application of the drug [7]. Thus, the inclusion of orally administered ivermectin in rotational deworming protocols against gastrointestinal helminths in elephants may be an alternative means of treating ear mites to avoid adverse events. Otitis in captive Asian elephants has been associated with the bacteria *Staphylococcus* sp. and *Pseudomonas* sp. [9], and potentially with *L. lenae* ear mites [7]. However, to our knowledge, the extent to which these organisms interact with other otitis-causing pathogens in Asian elephants has never been investigated. Therefore, the aim of this study was to assess the potential relationships between *L. lenae* ear mites, nematodes, yeast, bacterial rods, or cocci in the ears of Asian elephants under managed care in Thailand.

## Methods

### Animals

Legally owned, captive Asian elephants (*n* = 64; 13 males, 51 females), ranging in age from 11 months to 84 years, from seven herds (A–G), from six camps in Kanchanaburi Province and one camp in Chiang Mai Province, Thailand were sampled in this study (Table 1). The study population included six juveniles aged ≤ 15 years, 40 adults aged 16–50 years, 14 geriatric animals > 50 years old, and four animals of unknown age. Animals from herds A–F were sampled at the start of the rainy season (June–October), and the animals from herd G during the warm, dry season (February–May). Each animal underwent clinical assessment during routine preventative care as part of a larger investigation into elephant health.

**Table 1:** Number of elephants sampled from each herd according to sex and age class

Herd	Juveniles (≤ 15 years)		Adults (16–50 years)		Geriatric (> 50 years old)		Total
	Male	Female	Male	Female	Male	Female	
A	0	1	1	7	1	7	17
B	0	2	1	3	0	0	6
C	0	0	0	2	0	4	6
D	2	0	3	3	0	0	8
E	0	0	4	7	0	0	11
F	0	0	0	6	0	1	7
G	0	1	0	3	0	1	9^a^
Total	2	4	9	31	1	13	64^a^

^a^Total includes three females and one male of unknown age

### Sample collection and handling

Ear swab samples were obtained using non-sterile, 15-cm wooden, cotton-tipped applicators. Samples were collected from both ears of each individual, except for two individuals for which only the left ear was sampled, by gently lifting the muscular tragus surrounding the external auditory meatus, carefully inserting the swab 10–13 cm into the external ear canal and then removing it. Each cotton swab was then slowly rolled onto a glass slide for subsequent microscopy. Due to the difficulty of restraining the animals, we only sampled animals a second time if they were positive for mites or nematodes on microscopical examination. The second swabs were stored in 70% alcohol for morphological and molecular analysis.

### Sample analysis

Each slide was evaluated by light microscopy. The entire slide was examined using a ×40 objective to determine the presence and burden of nematodes and mites. Parasite burden was categorized as 0, 1+, or 2+ according to the presence of zero, from one to three, or four or more nematodes, and presence of zero, from one to five, or six or more mites per low power field, respectively (Table 2). Mites were identified by sex and biological stage using a DM-LB2 DIC microscope and Leica LAS version 4.5.0 software (Leica Microsystems, Wetzlar, Germany). Identification was carried out at up to ×100 magnification under oil immersion by comparing the morphologies of the mites found in the present study with those described for three ear mite species, namely, *Loxanoetus lenae* [6], *Loxanoetus* (*Auricanthus*) *longitarsus* [10], and *Loxanoetus bassoni* [11]. Five adult mites (stored in 70% alcohol) were selected from the samples and fixed overnight in 4% (volume/volume) glutaraldehyde in 0.0 M cold (4 °C) phosphate buffer at pH 7 and observed by low-pressure scanning electron microscopy (Hitachi TM3000). In addition, microscope slides were stained with Romanowsky stain and air dried to verify the presence and abundance of yeasts, bacterial rods, cocci, and host cells (i.e., epithelial cells, erythrocytes, and leukocytes) by light microscopy at ×100.

**Table 2:** Definitions of the categorical burdens of nematodes, mites, microorganisms (bacterial rods and cocci, yeasts), and host cells from microscopic evaluation of the ear swab samples

Organism/cell type	Category			
	0	1+	2+	3+
Nematodes	Absent	1–3/LPF	≥ 4/LPF	NA
Mites	Absent	1–5/LPF	≥ 6/LPF	NA
Microorganisms (bacterial rods/cocci, yeast)	Absent	Few organisms/HPF	Numerous organisms/HPF	Too numerous to count
Cells (leukocytes, epithelial cells)	Absent	1–5/LPF	≥ 6/LPF	NA

*LPF* Under low power field (×10) magnification,* /HPF* under high power field (×100) magnification, *NA* not applicable

Animals positive for mites were treated with a 1:1 dilution of saline and 1% injectable ivermectin solution (5 ml into each ear canal; 25 mg ivermectin per ear) by using a special applicator. Upon re-examination of ear cytology 1–3 days after application of the treatment, remnants of dead ear mites and nematodes were present on the slide, indicating that the parasites had been killed by the single treatment.

### Molecular analysis of mites

Prior to slide mounting, mite DNA was individually extracted by lysis with a guanidine isothiocyanate protocol, a method adapted from that described by Chomczynski [12]. Conventional polymerase chain reaction (PCR) was performed using primers that amplify a 480-base pair fragment of the V4 region of the 18S rRNA gene (18S+ and 18S−) [13]. The reactions were performed as follows: initial denaturation at 94 °C for 1 min, followed by 30 cycles of 94 °C for 20 s, 50 °C for 30 s and 72 °C for 1.5 min, with a final cycle with a lowering of the temperature to 25 °C. Amplicons were purified and sequenced using the Taq Dye Doxy Terminator Cycle Sequencing Kit (version 2; Applied Biosystems, CA) in an automated sequencer (ABI-PRISM 377). Sequences were analyzed by Geneious version 11.1.4 software and compared by Basic Local Alignment Search Tool (BLAST) with those available from the GenBank database.

### Molecular analysis of nematodes

DNA was individually extracted from 10 nematodes. The specimens were hand-picked and individually placed onto a glass slide in lysis buffer (10 mM Tris-HCl, pH8.8, 50 mM KCl, 15 mM MgCl_2_, 0.1% Triton X-100, 90 µg/ml proteinase K) and then cut into small pieces under a dissecting microscope. The samples were incubated at 65 °C for 1 h and then at 95 °C for 15 min to deactivate proteinase K. The PCR amplification, cloning and sequencing protocols have been described in detail by De Luca et al. [14]. The forward TW81 (5ʹ-GTTTCCGTAGGTGAACCTGC-3ʹ) and reverse AB28 (5ʹ-ATATGCTTAAGTTCAGCGGGT-3ʹ) primers were used to amplify the ITS1-5.8S-ITS2 region [15]; for the D2–D3 expansion segments of 28S rRNA, the forward D2A (5ʹ-ACAAGTACCGTGGGGAAAGTTG-3ʹ) and reverse D3B (5ʹ-TCGGAAGGAACCAGCTACTA-3ʹ) primers were used [16]; mitochondrial *cox*1 was amplified using COI-F1 (5ʹ-CCTACTATGATTGGTGGTTTTGGTAATTG-3ʹ) and COI-R2 (5ʹ-GTAGCAGCAGTAAAATAAGCACG-3ʹ) primers [17]. PCR amplifications were carried out in 100-µl volumes. The PCR mix [10 µl 10×PCR buffer, 2 µl dNTP mixture (10 mM each), 2 µl of each primer 10 mM, 0.25 µl of Taq DNA polymerase (Roche), 73.5 µl of distilled water and 10 µl of crude DNA] was added to each tube. The cycling conditions for the D2-D3 expansion domains and ITS region were as follows: initial denaturation at 94 °C for 7 min, followed by 35 cycles of denaturation at 94 °C for 50 s, annealing at 55 °C for 50 s, and extension at 72 °C for 50 s; the last step was at 72 °C for 10 min. For *cox*1, the conditions were as follows: initial denaturation at 94 °C for 3 min, followed by 45 cycles of denaturation at 94 °C for 30 s, annealing at 48 °C for 30 s, and extension at 72 °C for 30 s, and a final step at 72 °C for 7 min. PCR products were purified using the manufacturer’s protocol for the Nucleospin Extract II kit (Macherey–Nagel, Düren). The D2-D3 purified products were directly sequenced, while the ITS and *cox*1 products were cloned and sequenced in both directions with the M13 forward and M13 reverse primers. The pGEM-T Vector System II kit (Promega) was used to clone the PCR products.

A BLAST search of the new sequences using the National Center for Biotechnology Information (NCBI) database allowed us to identify the closest matching nematode taxonomic and/or phylogenetic groups. The newly obtained sequences were aligned with the most similar sequences of Rhabditida present in the database by using the Clustal W Omega program. Phylogenetic trees were reconstructed by the maximum likelihood (ML) method using MEGA11 software. A general time-reversible and gamma-shaped distribution model for D2-D3 domains and mitochondrial COI was used for the ML analysis. The phylograms were bootstrapped 1000 times to assess the degree of support for the phylogenetic branching indicated by the optimal tree for each method.

#### Statistics

Relationships between the presence and burden of nematodes and the presence and burden of mites, bacterial rods, bacterial cocci, yeast, leukocytes and epithelial cells from samples from the same and from contralateral ears were evaluated statistically. Burden was categorically defined for each variable (Table 2). Because of the small sample sizes, Fisher’s exact test was used for all comparative analyses using SAS (JMP Pro 15), including analysis of the presence of organisms or cell types between groups (i.e., sex, age class, and herd).

## Results

### Mites, nematodes, bacteria, and yeasts

Mites were detected in 43.8% (*n* = 28/64) of the animals (19 animals with mites in one ear and nine in both ears). Nematodes were detected in 23.4% (*n* = 15/64) of the animals (10 with nematodes in one ear and five with nematodes in both ears). In the cytology, cocci were detected in 85.9% (*n* = 55/64), rods in 70.3% (*n* = 45/64), and yeasts in 57.8% (*n* = 37/64) of the animals. On clinical examination, one geriatric female had signs of pruritus bilaterally, characterized by increased scratching of the head and ears on surrounding objects and the presence of abrasions and erythema on the pinnae and external ear canals. In addition, one juvenile male from the same herd exhibited signs of discomfort (vocalization and head shaking) during sample collection and had copious oily otorrhea from the right ear canal. The remaining animals were asymptomatic. Nematodes were more likely to be present in the ears of adult elephants than in those of the other age classes (Fisher’s exact test, two-tailed *P* = 0.0046), but nematode presence (Fisher’s exact test, two-tailed *P* = 0.8820) and burden (Fisher’s exact test, two-tailed *P* = 0.8820) did not differ between females and males. The detection of nematodes in either ear was positively associated with the detection of mites for adult elephants in general (Fisher’s exact test, two-tailed *P* = 0.0278), and for females (Fisher’s exact test, two-tailed *P* = 0.0107). There were no other significant differences between males and females or between any age class for any other parameter investigated. Nematodes (Fisher’s exact test, two-tailed *P* = 0.0157) and bacterial rods (Fisher’s exact test, two-tailed *P* = 0.018) were detected more frequently in elephants from herds F and G, and their presence varied significantly between herds. A higher categorical burden of nematodes in one ear was significantly associated with the presence of mites (Fisher’s exact test, two-tailed *P* = 0.0234) and epithelial cells (Fisher’s exact test, two-tailed *P* = 0.0108) in the same or contralateral ear, and was marginally significantly associated with the presence of bacterial cocci (Fisher’s exact test, two-tailed *P* = 0.0499) (Table 3).

**Table 3:** Associations of nematode presence and burden with mites, bacteria, yeast, and cells in the same ear and in the contralateral ear of the elephants

	Nematodes (present/absent)		Nematode burden (categorical)	
	Same ear	Contralateral ear	Same ear	Contralateral ear
Mites (±)	0.2734	0.0258*	0.0424*	0.0234*
Mites (category)	0.0117*	0.0007*	0.0005*	0.0023*
Rods	0.0073*	0.0074*	0.0017*	0.0523
Cocci	0.1051	0.0480*	0.0065*	0.0499
Yeast	0.3019	0.2114	0.0482*	0.2662
White blood cells	0.3509	0.3373	0.631	0.6471
Epithelial cells	0.0028*	0.0011*	0.0037*	0.0108*

* *P* ≤ 0.05 [Fisher’s exact test (two-tailed)]

### Mite identification

**Fig. 1:**
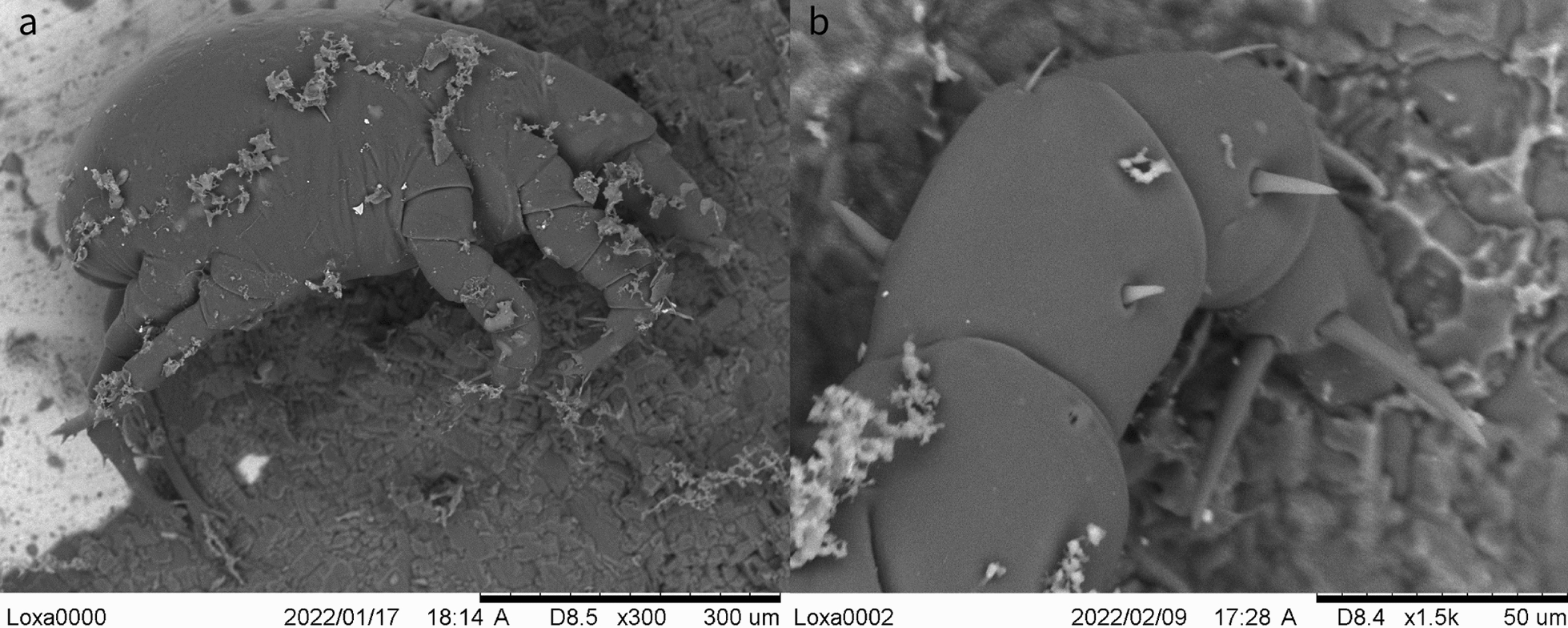
Scanning electron microscope images of the lateral view of the whole body and leg II of a *Loxanoetus lenae* Panels: Loxa0000 and Loxa0002 are the file names, followed by date and time in which the figure was prepared, the observation condition (Analysis mode), distance (mm), magnification, and reference scale bar

**Fig. 2:**
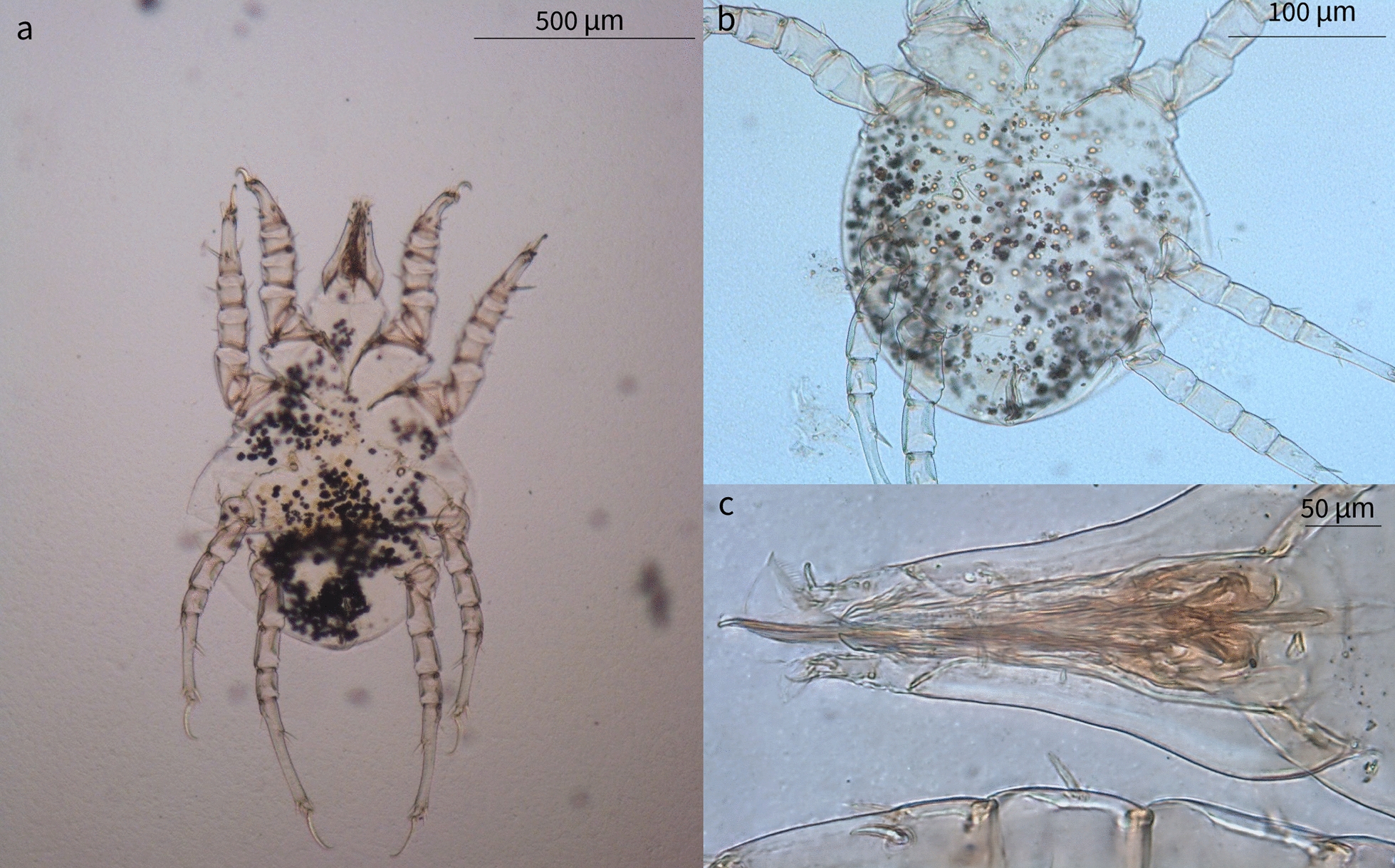
Ventral view (**a**), dorso-ventral view of the gnathosoma (**b**), and elongated capitulum (**c**) of an adult female *Loxanoetus lenae*

**Fig. 3:**
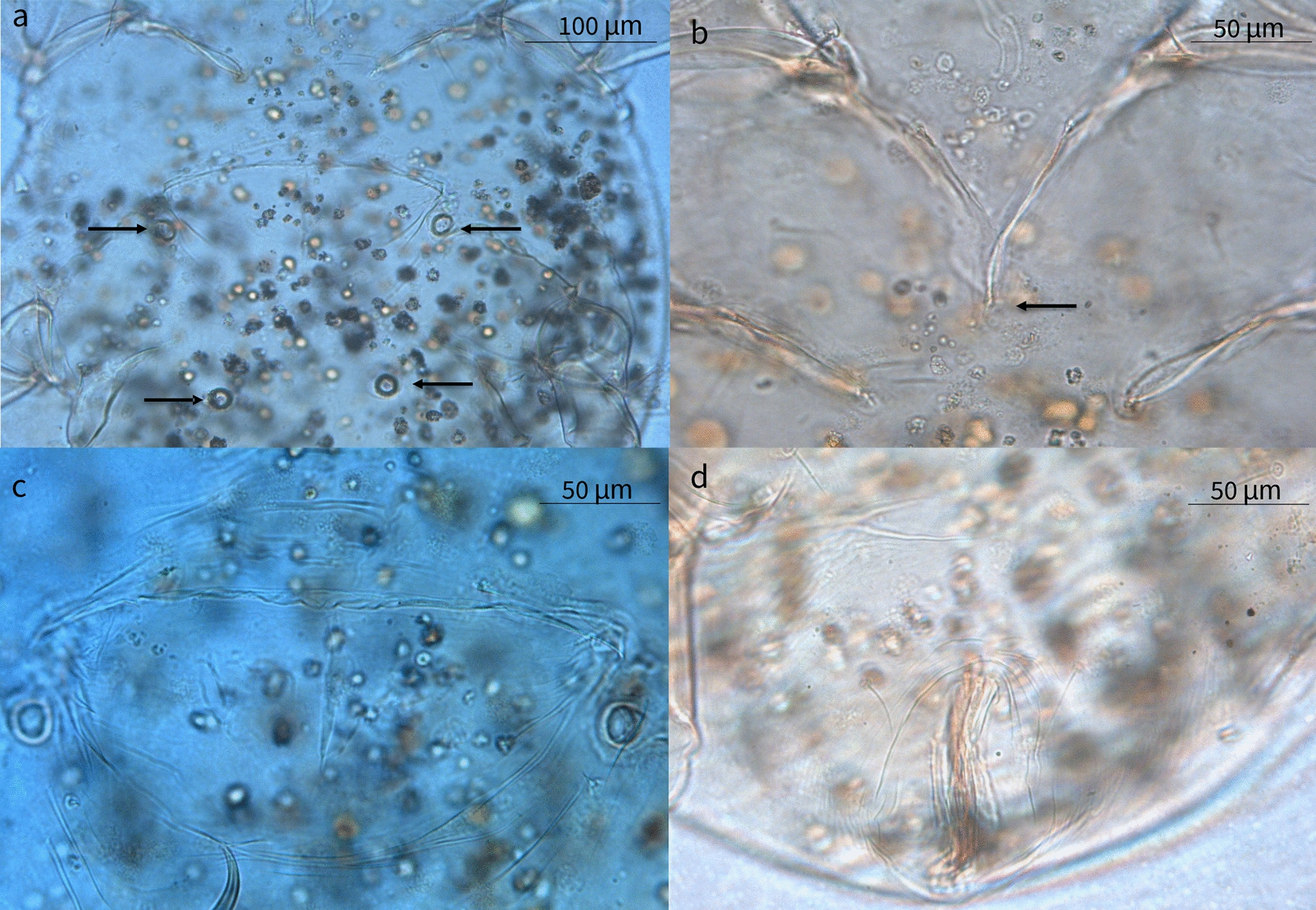
Two pairs of circular ventral rings (**a**), Y-shaped epimera I (**b**), genital aperture (**c**), and anal aperture (**d**) of an adult female *Loxanoetus lenae*

**Fig. 4:**
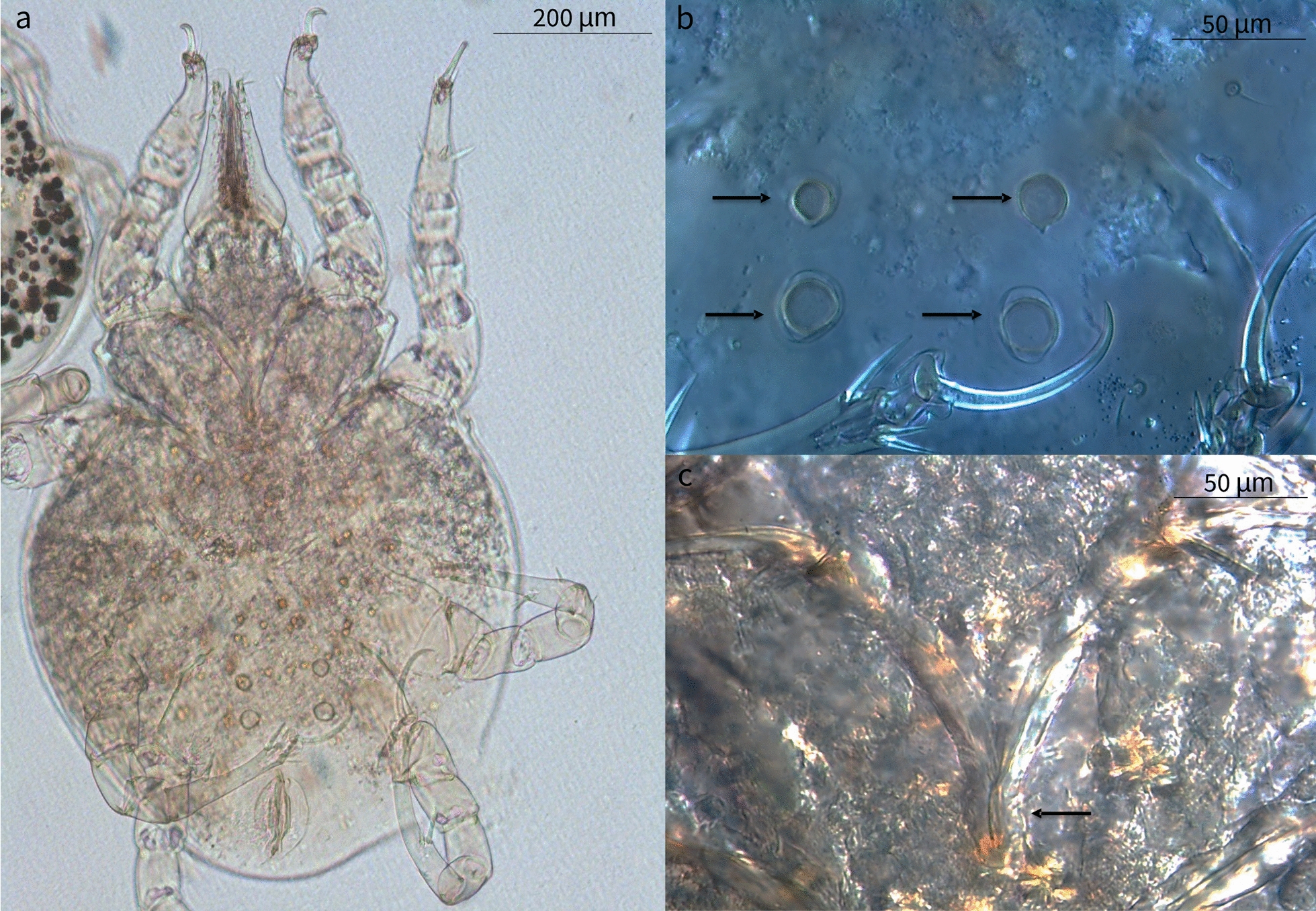
Ventral view of a tritonymph showing two pairs of ventral rings between coxae IV (**a, b**), and Y-shaped epimera I (**c**)

**Fig. 5:**
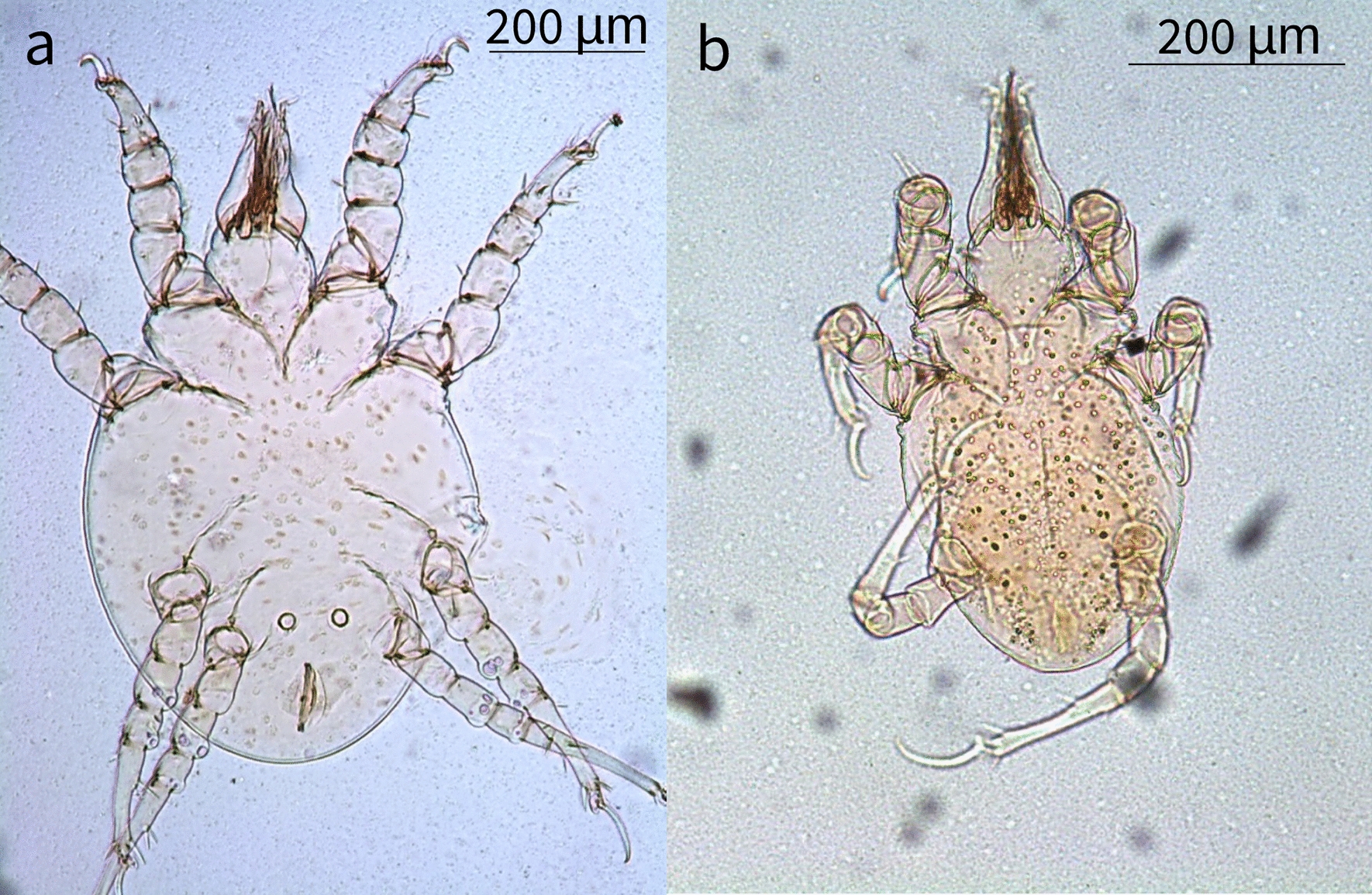
**a** Ventral view of a protonymph showing one pair of ventral rings between coxae IV. **b** A hexapodous first-stage larva

Larvae, protonymphs, tritonymphs, and adult mites were morphologically identified as *L. lenae* (Fig. 1). Adult mites were characterized by an elongated capitulum, two ventral elongated setae, a Y-shaped sternum (epimer I fused), a large genital opening consisting of a transverse slit that ends laterally at epimer III, and a well-developed ventral ring. A sejugal furrow trapezoidal in shape was present behind epimer II, and its sides reached the anterior ventral rings. The legs were robust with the tarsus presenting very strong spines and curved claws (short in legs I–II, and long and thin in legs III–IV). Midventral seta were present on tarsi III–IV along with two more, short, setae. Adult females presented an idiosoma that was 628.9 µm in length (SD ±45.03; range 556.7–661.1 µm) and 460.3 µm in width (SD ±9.4; range 450.4–475.8 µm), an elongated capitulum 203.1 µm in length (SD ±15.3; range 194.86–229.6 µm) (Fig. 2), a transverse genital aperture (57.1 µm long) that was membranous in appearance, and a transverse anal opening (Fig. 3). Adult males presented idiosoma that were 523.3 µm in length (SD ±55.4; range 523.4–658.8 µm) and 428.7 µm wide (SD ±64.3; range 404.7–537.6 µm), a capitulum 177.2 µm in length (SD ±7.6; range 167.0–188.0 µm), an oval anus aperture and small aedeagus with two clearly visible elongated processes. Tritonymphs had a capitulum that was 154.2 µm long (SD ±12.1; range 140.1–168.9 µm), and idiosoma that were 495.2 µm long (SD ±37.1; range 456.7–532.4 µm) and 349.5 µm wide (SD ±39.3; range 319.2–406.1 µm) (Fig. 4); protonymphs had a capitulum that was 146.3 µm long (SD ±6.6; range 138.4–167.5 µm) and idiosoma that were 465.3 µm long (SD ±58.0; range 420.1–559.3 µm) and 336.8 µm wide (SD ±54.5; range 282.8–421.4 µm) (Fig. 5a); and larvae had a capitulum that was 129.2 µm long (SD ±8.5; range 126.4–137.7 µm) and idiosoma that were 334.5 µm long (SD ±13.6; range 318.0–350.2 µm) and 220.5 µm wide (SD ±3.2; range 218.1–225.4 µm) (Fig. 5b). The chaetotaxy of the podosoma and hysterosomal dorsal–ventral setae are reported in Table 4.

**Table 4:** Morphological comparison of female mites from the ears of Asian elephants examined in the present study with three species of ear mites of the family Histiostomatidae; for chaetotaxy and abbreviations, see Atyeo and Gaud [18, 19]

Observations	*Loxanoetus* *lenae*	*Loxanoetus* *longitarsus*	*Loxanoetus* *bassoni*	This study
Dorsal propodosomal setae				
vi	Absent	Vestigial	Absent	Absent
ve	Absent	Vestigial	Absent	Absent
sci	Present	Present	Present	Present
sce	Present	Present	Present	Present
Dorsolateral setae				
h	Present	Present	Present	Present
sh	Present	Present	Present	Present
d	2, 3, 4, 5	4, 5	2, 3, 4, 5	2, 3, 4, 5
l	2, 3, 4, 5	1, 2, 3, 4, 5	1, 2, 3, 4, 5	2, 3, 4, 5
Ventral setae				
c1 (ga)^a^	Present	Present	Present	Present
c2 (gm)^a^	Present	Present	Present	Present
c3 (gp)^a^	Present	Present	Present	Present
a (ps3)	Present	Present	Present	Present
Coxal setae				
cxl	Present	Present	Present	Present
cxll	Absent	Absent	Absent	Absent
cxlll	Present	Present	Present	Present
cxIV	Absent	Absent	Absent	Absent
Solenidia on tarsi				
ω1	ω1, ω2, ω3	ω1, ω2, ω3	ω1, ω2	ω1, ω2, ω3
ω2	ω1		ω1	ω1
ω3	Absent		Absent	Absent
ω4	Absent		Absent	Absent
Solenidia on tibia				
φ1	Φ1		Φ1	Φ1
φ2	Φ1		Φ1	Φ1
φ3	Φ1		Φ1	Φ1
φ4	Φ1		Absent	Φ1
Solenidia on genu				
σ1	σ1, σ2	σ1	σ1	σ1, σ2
σ2	σ1	Absent	Absent	σ1
σ3	Absent	Absent	Absent	Absent
σ4	Absent	Absent	Absent	Absent
Solenidia on femur				
θ1	Absent	Absent	Absent	Absent
θ2	Absent	Absent	Absent	Absent
θ3	Absent	Absent	Absent	Absent
θ4	Absent	Absent	Absent	Absent
Chaetotaxy				
Coxa	1.0.1.0	1.0.1.0	1.0.1.0	1.0.1.0
Trochanter	1.1.1.0	1.1.1.0	1.1.1.0	1.1.1.0
Femur	1.1.0.1	1.1.0.1	1.1.0.0	1.1.0.1
Genu	2.2.0.0	2.2.0.0	2.2.0.0	2.2.0.0
Tibia	2.2.1.1		2.2.1.0	2.2.1.1
Tarsus	13.12.10.10	12.12.10.10	12.12.10.10	13.12.10.10

^a^Modified from Fain [20, 21]

In the molecular analysis, the amplified products showed 93% similarity with mite sequences of the family Histiostomatidae.

### Nematode identification

**Fig. 6:**
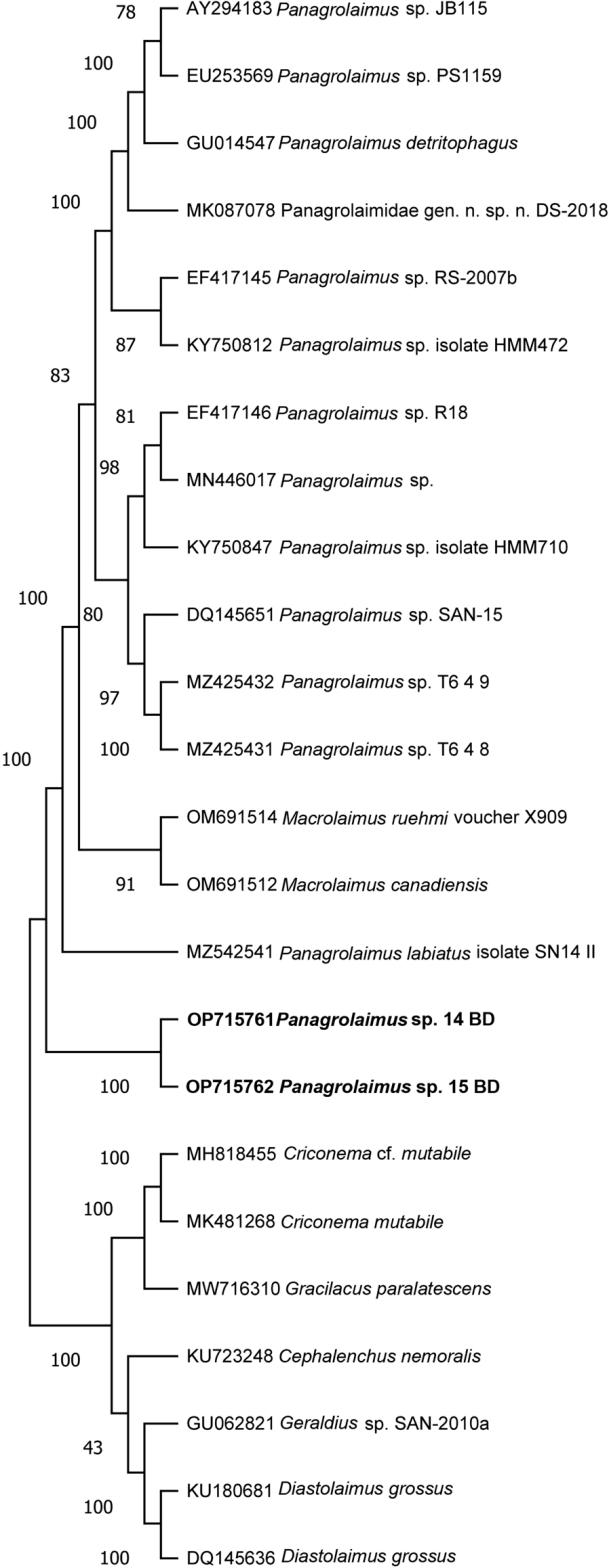
Phylogenetic tree of D2–D3 expansion domains of the 28S rRNA gene describing the evolutionary relationships among different *Panagrolaimus* species, determined using the maximum likelihood method. Branch lengths are proportional to the distances derived from the distance matrix obtained using the general time-reversible method with the invariant site plus gamma options. Numbers at nodes indicate bootstrap values. Newly obtained sequences from this study are in bold

**Fig. 7:**
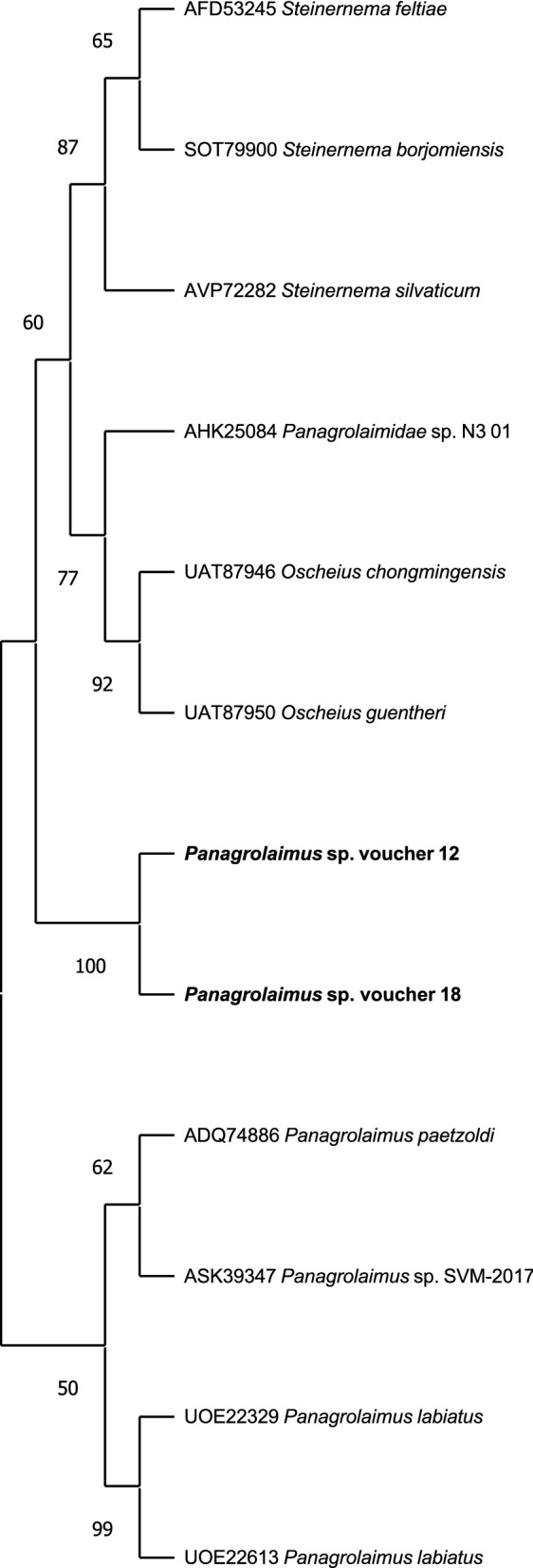
Phylogenetic tree of partial mitochondrial *cox*1 sequences describing the evolutionary relationships among different *Panagrolaimus* species, determined using the maximum likelihood method. Branch lengths are proportional to the distances derived from the distance matrix obtained using the general time-reversible method with the invariant site plus gamma options. Numbers at nodes indicate bootstrap values. Newly obtained sequences from this study are in bold

Molecular analysis of the nematodes showed no intra-population sequence variability in the D2–D3 sequences. A BLAST search showed 88% similarity of the D2–D3 region with the corresponding region of several *Panagrolaimus* spp. and Rhabditida present in the GenBank database. The D2–D3 alignment included 29 sequences, and phylogenetic relationships among the closest species revealed that the nematode population isolated from Thailand clustered into a well-supported group (100% support) of other *Panagrolaimus* populations from the database (Fig. 6). BLAST search at NCBI of the ITS sequence of the Bangladesh nematode population revealed no corresponding sequences in the database. The *cox*1 of two specimens were cloned, and three clones were sequenced for the first time in the present study. BLAST search of the NCBI database revealed 92–90% similarity at the amino acid level with *Panagrolaimus* spp. in the database. The phylogenetic tree revealed that the Thailand nematode population clustered with *Panagrolaimus* populations with 67% of support (Fig. 7). Newly obtained sequences were deposited in GenBank under accession numbers OP715761–OP715762 for D2–D3 and OP720885–OP720886 for the *cox*1 sequences.

## Discussion

We report the association of nematodes, *L. lenae* ear mites, bacteria, and yeasts that had colonized the external ear canals of Asian elephants. Of note, a higher frequency of nematodes was observed in individuals positive for ear mites and/or bacterial rods, with a higher intensity of nematodes when *L. lenae* mites were present. This association could be due to the fact that ear mites cause otic discomfort, which may lead to an increase in dust-bathing behaviors and therefore an increased risk of the incidental introduction of geohelminths into the ear. Dust-bathing behavior is important in regulating body temperature, protecting the skin from sunlight, protecting against parasitic infections, or a combination of these factors [22]; however, we suggest that an increased frequency of dust-bathing could negatively impact the health of elephants by introducing incidental pathogens into the ear canal. Mites of the genus *Loxanoetus* have been previously described in these large mammals, with the species *L. bassoni* described in African elephants and *L. lenae* in Asian elephants [3, 6]. Both species are found in the ear, but their association with other causative agents of otitis has never been previously investigated in a large captive population of elephants, to the best of our knowledge. In addition, clinical signs were also observed in two out of the 28 positive animals (i.e., bilateral pruritus, increased scratching of the head and ears on surrounding objects, the presence of abrasions and erythema on the pinnae and external ear canals, vocalization and head shaking during sample collection, and oily otorrhea in the right ear canal). The fact that most of the positive animals (i.e., 26 out of 28) were apparently asymptomatic shows the importance of performing ear swab cytology in elephants to determine the presence of ear mites, nematodes, rods or yeasts as part of routine physical examinations, and to treat appropriately if these organisms are detected. The high prevalence (i.e., 43.8%) of ear mites in the studied population may also be a result of direct horizontal transmission of *L. lenae* among animals confined to the same environment. However, to our knowledge, there are no published data on the transmission routes of ear mites in elephants, which indicates a need for further studies on the biology and epidemiology of these ectoparasites.

The phylogenetic relationships based on the D2–D3 and mitochondrial COI sequences indicated that the nematodes collected from the elephant ears belong to the genus *Panagrolaimus*. Parasites of the genus *Panagrolaimus* are soil-borne and widely distributed globally, and inhabit several types of environment due to their ability to show diverse adaptations or cryptobiosis [23, 24]. However, whether they cause ear infections in elephants remains to be clarified.

There are only a few reports of clinical otitis in elephants in the scientific literature. This may be due to the difficulty of performing satisfactory otic examination of these animals, since they need to be physically or chemically restrained or trained for voluntary behavioral restraint, and the tools or methods for this are not always available (e.g., improved restraint and handling tools, drugs and methods for their administration, and training for operant conditioning), as reported by veterinarians in a previous survey on the health and management of captive Asian elephants [25]. It may also be possible to train captive elephants through protected contact with positive reinforcement to allow ear sample collection. Among the few published reports of otitis in Asian elephants, clinical signs were reported for a 50-year-old elephant that presented with purulent discharge from the left ear canal which was associated with the presence of *Staphylococcus* spp. and *Pseudomonas* spp. [9]. However, the significant association, or co-infection, between nematodes, bacterial rods, and/or ear mites reported in the present study indicates the need for further research to confirm the role of parasites as causative agents of otitis in elephants.

## Conclusions

The results of the present study suggest that the presence of *L. lenae* mites in the ear canal of Asian elephants is significantly associated with the occurrence of other microorganisms, such as soil nematodes, bacteria and yeasts. In addition, we suggest that the presence of mites may increase dust-bathing behavior in infested animals and, therefore, the amount of dust introduced into their ears, which in turn favors the entry of geohelminths and other microorganisms into the ear canal.

## Data Availability

The authors declare that the data supporting the findings of this study are available within the article. Sequences of *L. lenae* have been deposited in GenBank (accession number OP995483).

## Abbreviations

BLAST: Basic Local Alignment Search Tool
ML: Maximum likelihood
NCBI: National Center for Biotechnology Information
PCR: Polymerase chain reaction
